# A Low-Noise and High-Integration Readout IC with Pixel-Level Single-Ended CDS for Short-Wave Infrared Focal Plane Arrays

**DOI:** 10.3390/s26030847

**Published:** 2026-01-28

**Authors:** Hongyi Wang, Songlei Huang, Zhenghua Peng, Song Jing, Runze Xia, Yu Chen, Panjie Dai, Jiaxiong Fang

**Affiliations:** 1 National Key Laboratory of Infrared Detection Technologies, Shanghai Institute of Technical Physics, Chinese Academy of Sciences, Shanghai 200083, China; wanghongyi@mail.sitp.ac.cn (H.W.); pengzhenghua23@mails.ucas.ac.cn (Z.P.); jingsong@mail.sitp.ac.cn (S.J.); xiarunze@mail.sitp.ac.cn (R.X.); chenyu@mail.sitp.ac.cn (Y.C.); daipanjie@mail.sitp.ac.cn (P.D.); jxfang@mail.sitp.ac.cn (J.F.); 2 State Key Laboratories of Transducer Technology, Shanghai Institute of Technical Physics, Chinese Academy of Sciences, Shanghai 200083, China

**Keywords:** ROIC, short-wave infrared focal plane arrays, pixel-level correlated double sampling, low-noise design, capacitor-reuse technique, integration-while-read

## Abstract

Improving sensitivity in short-wave infrared (SWIR) detection is crucial for low-signal applications, such as astronomy and hyperspectral imaging, which demand readout integrated circuits (ROICs) with minimal noise and high density. However, conventional differential pixels with correlated double sampling (CDS) are difficult to integrate due to spatial limitations. In order to tackle this issue, we propose a compact, pixel-level, single-ended charge-domain architecture. It integrates single-ended CDS within each pixel, guaranteeing compatibility with the integrate-while-read (IWR) mode while suppressing reset and 1/*f* noise. A capacitor reuse technique is also proposed to enable the integration capacitor to function as an auxiliary load, which optimizes the noise–area trade-off. Fabricated in 180 nm CMOS, our 1296 × 256 ROIC attains a noise floor of 0.50 mV (achieving a reduction of approximately 70% compared to conventional architectures under identical conditions), consumes under 200 mW, and operates at frequencies exceeding 200 Hz. It also exhibits great linearity (0.9999) and supports both integrate-then-read (ITR) mode and integrate-while-read (IWR) mode, while also providing a row-level gain selecting function. Validated at 15 μm pitch, this design provides an effective option for high-density SWIR systems.

## 1. Introduction

Short-wave infrared (SWIR) imaging can penetrate smoke and haze and identify materials, which makes it valuable for night vision, remote sensing, astronomical observation, and biomedical imaging [1]. The demand for higher resolution in imaging has reduced the pixel pitch of infrared focal plane arrays (FPAs) to 15 µm or less, resulting in significant design issues for readout integrated circuits (ROICs) [2]. As the central element in a detection system, the ROIC’s noise level, integration density, and power efficiency fundamentally determine the system’s performance [3]. Therefore, enabling ultra-low-noise readout within such a limited pixel area has become a primary challenge for advancing SWIR imaging.

Noise in readout circuits, particularly reset (KTC) noise and low-frequency 1/*f* noise, constrains the sensitivity and dynamic range of infrared detectors, which makes noise reduction a priority in readout circuit design [4]. Correlated double sampling (CDS) is a widely adopted solution for this purpose in high-performance ROICs [5]. Meanwhile, the integrate-while-read (IWR) mode has become essential for high-frame-rate applications, as it improves integration efficiency. However, IWR’s readout switching processes cause significant additional noise [6]. This presents a design dilemma: the successful integration of both CDS and IWR functions within a very constrained pixel region is essential yet incredibly difficult.

Current approaches face challenges in overcoming this issue. For instance, the traditional differential-output architecture requires independent signal and reset sampling paths. This requirement substantially increases chip area and power consumption, ultimately proving incompatible with stringent pixel size constraints [7]. Column-level CDS techniques conserve pixel area but cause their own trade-offs: complex timing control can lead to column fixed-pattern noise and increased power consumption. Conventional single-ended outputs are more area-efficient but typically lack full functionality; they normally cannot support effective CDS or remain compatible with the IWR mode [8]. These constraints significantly impede the advancement of high-resolution, compact SWIR detectors [9].

Compared to traditional pixel-level differential CDS architectures, we propose a pixel-level compact single-ended CDS architecture. It dispenses with the dual independent sampling paths and capacitors found in conventional approaches, instead employing time-delay sampling and polarity switching techniques to implement CDS functionality within a single path. Compared to column-level CDS architectures, this work fully integrates CDS functionality within the pixel itself. This eliminates the need to transmit reset signals and signal levels via column buses to column-level circuits for processing, thereby avoiding fixed-pattern noise introduced at the column level. Furthermore, this structure achieves efficient compatibility between CDS and IWR modes within the pixel, enabling high-frame-rate output from the circuit. The introduction of an isolated operational amplifier resolves signal integrity issues arising from charge redistribution within the single-ended path. Furthermore, the proposed capacitance multiplexing technique dynamically increases the equivalent load capacitance without adding extra area, optimizing the noise-to-area trade-off and achieving low readout noise in IWR mode.

In this paper, we propose a novel pixel-level, single-ended CDS architecture that successfully co-integrates full CDS functionality with IWR operation at 15 µm pitch. This design directly tackles the core trade-off between noise performance and integration density in scaled pixels. Based on this architecture, we design and fabricate a 1296 × 256 ROIC prototype using a standard 180 nm CMOS process. Test results indicate that this circuit chip achieves approximately 70% noise reduction in IWR mode compared to conventional architectures under identical conditions. The circuit noise voltage is 0.5 mV, and it exhibits excellent linearity of 0.9999. The overall power consumption is below 200 mW, the highest readout frame rate exceeds 200 Hz, and the system accommodates both IWR and ITR working modes. These results validate the technical viability and practical value of our approach, providing a crucial foundation for next-generation, high-resolution, and low-power SWIR imaging systems.

## 2. Infrared Focal Plane Array

### 2.1. IRFPA Architecture

**Figure 1:**
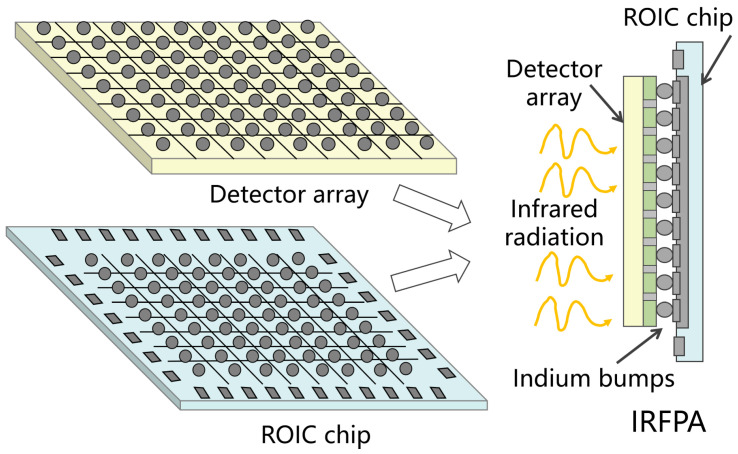
Infrared focal plane array (IRFPA) architecture.

Infrared focal plane arrays (IRFPAs) are the core components of modern infrared imaging systems, responsible for converting incoming infrared radiation into electrical image signals. As illustrated in Figure 1, a typical IRFPA features a hybrid architecture. It consists of two main parts: a photosensitive detector array and a readout integrated circuit (ROIC). In this structure, the detector array is flip-chip bonded to the underlying ROIC via indium bump interconnections, forming the complete focal plane assembly.

The detector array captures incoming infrared radiation and converts it into photocurrent. The readout integrated circuit (ROIC) then performs several key functions. It supplies bias voltage to the detector array. It also processes the resulting signal. This processing includes signal conversion, amplification, and multiplexed readout. Internally, the ROIC contains two main parts: digital control logic and analog circuit modules [10]. The analog modules are the performance-critical core. They define key parameters, such as charge handling capacity, output swing, and signal-to-noise ratio. These modules execute essential operations such as integration reset, sample-and-hold, and sequential shift-readout. Their role is to relay the weak signals from each detector pixel to the output port in sequence. As the critical enabling technology for infrared focal plane arrays, the ROIC’s performance is paramount. It ultimately determines the final image quality of the entire IRFPA system [11].

### 2.2. ROIC Chip Architecture

**Figure 2:**
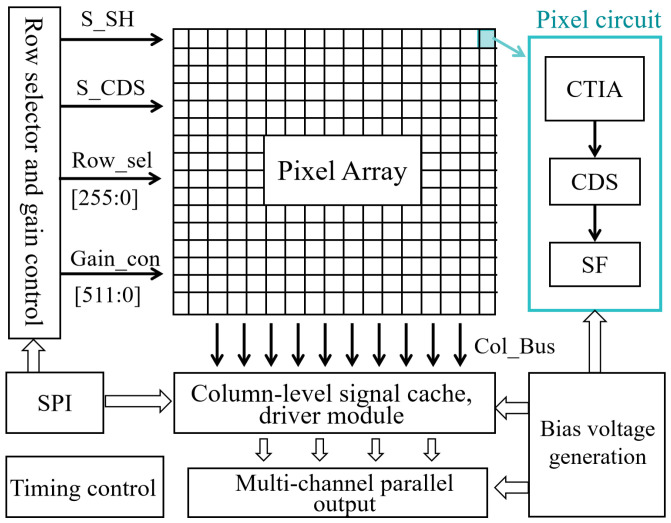
The overall structure of ROIC.

Figure 2 shows the architecture of the readout integrated circuit (ROIC) chip, which contains four main modules: a serial peripheral interface (SPI), a row-select controller, an analog pixel array, and column-level output buffers. The analog pixel circuit integrates three key stages: a capacitive transimpedance amplifier (CTIA) input stage, a correlated double sampling (CDS) circuit, and a source follower (SF) [12]. Among these, the CTIA input stage is the preferred front-end for short-wave infrared focal plane arrays. It provides a stable bias for the photodetectors and offers excellent linearity, a wide dynamic range, and low noise. The following CDS stage effectively suppresses KTC reset noise.

After sampling, the signal is buffered by a pixel-level amplifier and driven onto the column bus. It then undergoes further amplification at the column level before being sequentially read out through parallel output channels. This process is controlled by row-select and column-select signals. The SPI module enables programmable control. It manages row/column selection and configures multi-step gain for each row through specific command words.

### 2.3. Conventional Pixel-Level CDS

Correlated double sampling (CDS) is a key technique for achieving low-noise readout in infrared focal planes. It works by taking the difference between the integrated signal level and a reset level, effectively suppressing reset (KTC) noise, low-frequency 1/*f* noise, and amplifier thermal noise [13]. Based on its location in the readout chain, CDS is implemented at either the pixel or column level. For short-wave infrared detectors operating in global shutter snapshot mode, high frame rates are essential. This requires an integrate-while-read (IWR) operation scheme [14]. IWR, in turn, needs a signal storage unit within each pixel. Consequently, a pixel-level CDS architecture becomes the natural choice.

**Figure 3:**
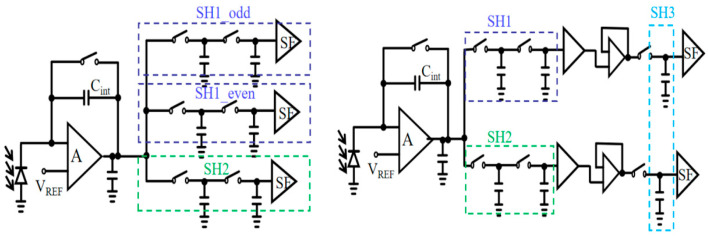
Pixel-level differential output CDS architecture for traditional IWR readout.

Figure 3 shows a conventional pixel-level CDS design. It requires two separate sets of sampling switches and capacitors to independently sample the reset and signal levels. To also support IWR operation, additional switching transistors and storage nodes must be added. This increases the complexity of control logic and routing congestion, significantly expanding the pixel area [5]. This area overhead makes it difficult to scale the pixel pitch below 20 µm in traditional designs, limiting their application in high-resolution, small-pixel detectors [15].

## 3. Low-Noise Pixel Circuit Design

### 3.1. Pixel-Level Single-Ended CDS

**Figure 4:**
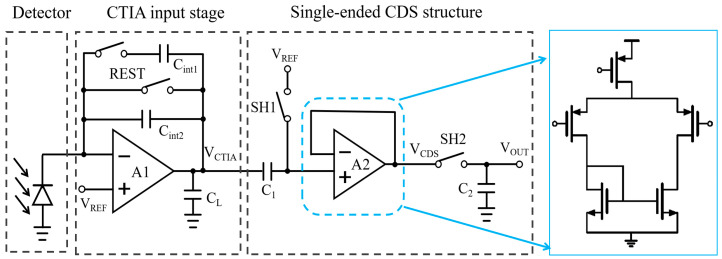
Pixel-level single-ended CDS circuit architecture.

We propose a novel, compact pixel-level single-ended CDS architecture suitable for narrow pixel pitches (Figure 4). Its core innovation is a time-delayed sampling technique. This technique performs differential sampling of the reset and signal levels progressively through a single signal channel. The final differential voltage is stored on just one sampling capacitor (C_2_). This approach delivers full CDS functionality within one frame cycle while drastically reducing the pixel area. To preserve signal integrity, we integrate an isolating amplifier (A_2_). It prevents interference from capacitor charge redistribution during sampling, thereby maintaining the output signal amplitude. The core function of operational amplifier A_2_ is to serve as a voltage buffer, necessitating high-impedance inputs and low-impedance outputs. As illustrated in Figure 4, given stringent pixel area constraints, A_2_ employs a high-linearity, simplified operational amplifier configuration. Furthermore, A_2_ utilizes negative feedback to form a voltage follower, realizing a unity-gain voltage buffer that delivers the requisite isolation and drive capability with minimal area overhead. Moreover, as A_2_ resides within the sampling path, it sequentially samples both reset levels and signal levels. Consequently, any noise introduced by non-ideal factors such as op-amp input offset within the differential output is fully canceled. This ensures it does not compromise the core mechanism of CDS suppression against related noise. For signal subtraction, the circuit uses polarity switching across the capacitor. This design optimizes area efficiency to fit within strict pixel dimensions. Furthermore, the single-ended output architecture simplifies the chip’s overall I/O design. It also helps lower power consumption in the column-level readout circuitry.

**Figure 5:**
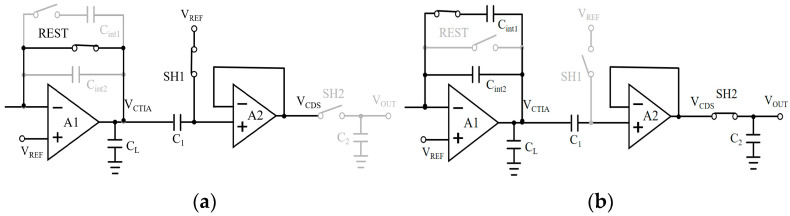
(**a**) Schematic diagram of equivalent circuit connections during the reset phase. (**b**) Schematic diagram of equivalent circuit connections during the signal sampling phase.

The proposed compact single-ended CDS architecture operates across four consecutive phases within a complete working cycle: the reset phase, integration phase, sampling phase, and readout phase. During the reset phase, switches REST and SH1 are closed while SH2 is open, with the equivalent circuit structure depicted in Figure 5a below. At this point, the integration capacitors C_int1_, C_int2_, and C_1_ are reset to the reference voltage *V*_REF_, clearing their stored charge capacities. The voltage at the CTIA output node *V*_CTIA_ is *V*_REF_. During the integration phase, the REST switch opens, and the CTIA circuit begins integrating the photocurrent. The voltage at the output node *V*_CTIA_ linearly decreases from *V*_REF_. During this period, SH2 remains open. The reset level sampling switch SH1 opens after a brief delay following the start of integration to sample the valid reset level. Upon SH1 opening, the instantaneous voltage is captured and held across capacitor C_1_. During this phase, the voltage at C_1_’s right plate linearly decreases in line with the *V*_CTIA_ signal, transmitted via the unity-gain buffer A_2_ to node *V*_CDS_. Throughout the sampling phase, SH1 remains open. Following the preset integration time, SH2 closes. The equivalent circuit configuration at this point is illustrated in Figure 5b. The upper plate of sampling capacitor C_2_ now samples and holds the node voltage at *V*_CDS_. During the readout phase, switch SH2 opens. The voltage stored across capacitor C_2_ at this point represents the difference between the sampled signal and the reset signal. This differential voltage is directly output via the output terminal, thereby directly implementing the relevant dual-sampling functionality.

**Figure 6:**
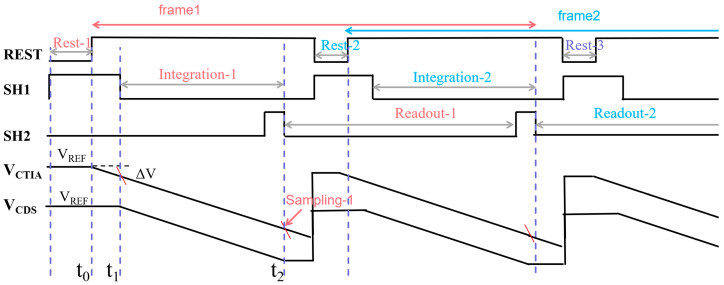
The integrate-while-read (IWR) read timing diagram.

As illustrated in Figure 6, the proposed compact single-ended CDS architecture demonstrates its operational timing diagram. Due to the parallel execution of integration and readout phases between adjacent frames, this circuit operates in an integrated-while-reading (IWR) mode. Here, t_0_ is defined as the moment when the reset phase concludes and integration commences. t_1_ denotes the reset level sampling instant, deliberately delayed until after integration commences to ensure capacitor C_1_ samples a stable reset level. t_2_ marks the signal-level sampling instant, coinciding with the conclusion of integration. At this point, the circuit’s differential signal is output via the isolation amplifier A_2_, directly implementing the relevant dual-sampling functionality. It should be noted that to ensure orderly readout in IWR mode, the readout cycle of the current frame must conclude before the SH2 sampling switch of the subsequent frame closes.

During the integration phase, the output voltage of the CTIA can be expressed as

(1) $$V_{\mathrm{CTIA}}(t)=V_{\mathrm{REF}}-\frac{I_{d}\left(t-t_{0}\right)}{C_{\mathrm{int}}},$$

where *V*_CTIA_(*t*_0_) = *V*_REF_. Meanwhile, the voltage at the *V*_CDS_ node satisfies

(2) $$V_{\mathrm{CDS}}=\left\{\begin{array}{c} V_{\mathrm{REF}},\;\;t<t_{1}\\ V_{\mathrm{REF}}-\left[V_{\mathrm{CTIA}}(t_{1})-V_{\mathrm{CTIA}}(t)\right],\;\;t_{1}\leq t\leq t_{2}\\ V_{\mathrm{CTIA}}(t_{2})+\Delta V,\;\;t>t_{2} \end{array}\right.,$$

Based on the principle of charge conservation, the following relationship can be established:

(3) $$\Delta V=V_{\mathrm{CTIA}}(t_{0})\;-{\;V}_{\mathrm{CTIA}}\left(t_{1}\right),$$

When *t* > *t*_2_, the above expression can be simplified to

*V*_CDS_ = *V*_REF_ + [*V*_CTIA_(*t*_2_) − *V*_CTIA_(*t*_1_)]. (4)

**Figure 7:**
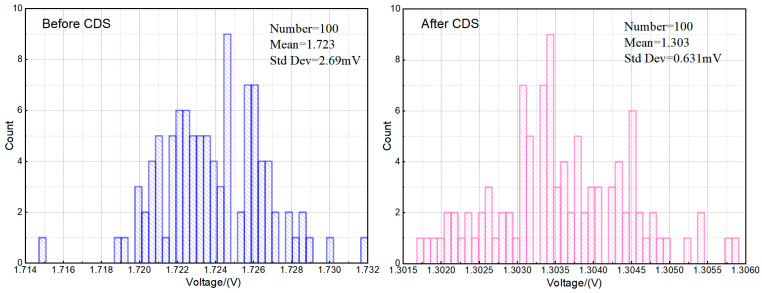
Comparison of transient noise simulation results before and after the CDS circuit.

This result shows a clear relationship: when sample switch SH_2_ turns on at time *t*_2_, the pixel’s final output equals the voltage difference between the reset and signal levels. This confirms the successful implementation of correlated double sampling (CDS) functionality within the circuit. The transient noise simulation outcomes of the circuit, depicted in Figure 7 below, confirm the effectiveness of this function: The circuit noise voltage decreases from 2.69 mV before CDS processing to 0.631 mV after processing, illustrating the enhanced noise suppression efficacy of this single-ended CDS architecture.

### 3.2. ROIC Noise Analysis

The total noise of an infrared focal plane array (IRFPA) mainly comprises detector chip noise, readout integrated circuit (ROIC) noise, and coupling noise at their interface [4]. The primary sources of noise affecting the ROIC encompass thermal noise from the input-stage operational amplifier (including coupling interference with the detector), KTC noise from the reset switch, sampling circuit noise, output-stage noise, and 1/*f* noise from MOS devices [16]. The formulations for each noise source are presented as follows [17]:

(5) $$\sigma_{\mathrm{AMP}}=\left(C_{\mathrm{int}}+C_{\det}\right)\sqrt{4\alpha \mathrm{kT}C_{\mathrm{int}}/C_{x}}/q,$$

(6) $$\sigma_{\mathrm{RST}}=\sqrt{kT\left(C_{\mathrm{int}}+C_{\det}\left(C_{L}+2\alpha C_{\det}\right)C_{\mathrm{tot}}\right)}/q,$$

(7) $$\sigma_{\mathrm{SH}}=C_{\mathrm{int}}\sqrt{2\mathrm{kT}/C_{\mathrm{SH}}}/q,$$

(8) $$\sigma_{\mathrm{SF}}=C_{\mathrm{int}}\sqrt{8\mathrm{kT}/\pi g_{m,\mathrm{SF}}R_{\mathrm{out}}C_{\mathrm{out}}}/q,$$

where *C*_int_ is the integration capacitance, *C*_det_ is the detector capacitance, *C*_L_ is the load capacitance of the readout circuit, *C*_SH_ is the sampling capacitance, *C*_tot_ = *C*_int_ + *C*_det_ + *C*_L_, *C*_x_ = *C*_int_*C*_det_ + *C*_det_*C*_L_ + *C*_L_*C*_int_, *α* = 1.3, *k* = 1.38 × 10^−23^ J/K, *T* = 300 K, and *q* = 1.6 × 10^−19^ C. Here, $\sigma_{\mathrm{AMP}}$ denotes the operational amplifier thermal noise, $\sigma_{\mathrm{RST}}$ represents the KTC noise, $\sigma_{\mathrm{SH}}$ is the sampling noise, and $\sigma_{\mathrm{SF}}$ corresponds to the output-stage noise.

The correlated double sampling (CDS) architecture employed in the readout circuit effectively mitigates KTC noise and the low-frequency 1/*f* noise of the amplifier, enabling the exclusion of these two components from the noise analysis. Assuming the noise sources are mutually uncorrelated, the total noise of the readout circuit can be expressed as

(9) $$\sigma_{\mathrm{ROIC}}=\sqrt{{\sigma_{\mathrm{AMP}}}^{2}+{\sigma_{\mathrm{SH}}}^{2}+{\sigma_{\mathrm{SF}}}^{2}}$$

**Figure 8:**
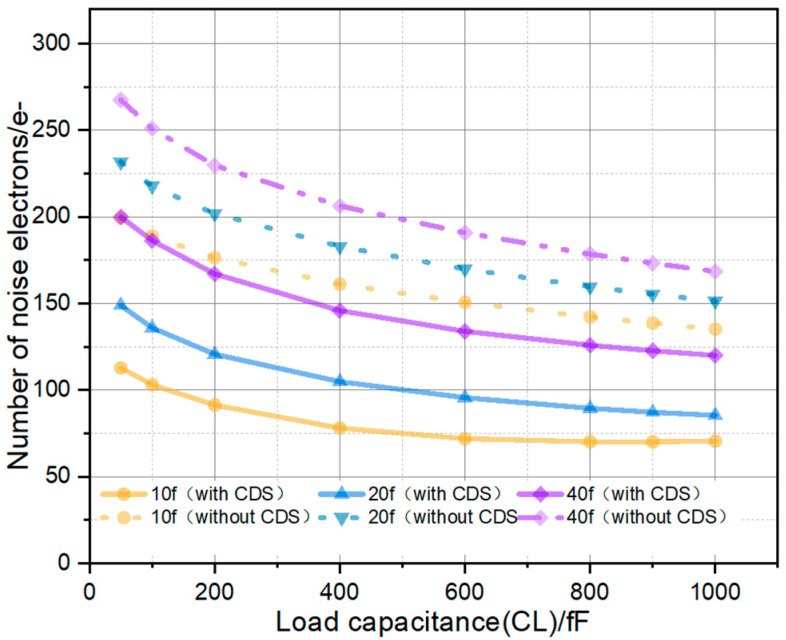
Simulation of the effects of integral capacitor (*C*_int_) and load capacitor (*C*_L_) on noise.

It can be deduced from the aforementioned formulations that diminishing the integration capacitance *C*_int_ and the detector junction capacitance *C*_det_, while augmenting the load capacitance *C*_L_, facilitates a reduction in readout noise. Figure 8 below displays static noise simulation results that demonstrate the effect of integration and load capacitances on noise performance, given a constant detector junction capacitance (*C*_det_).

### 3.3. Capacitor Reuse Architecture

**Figure 9:**
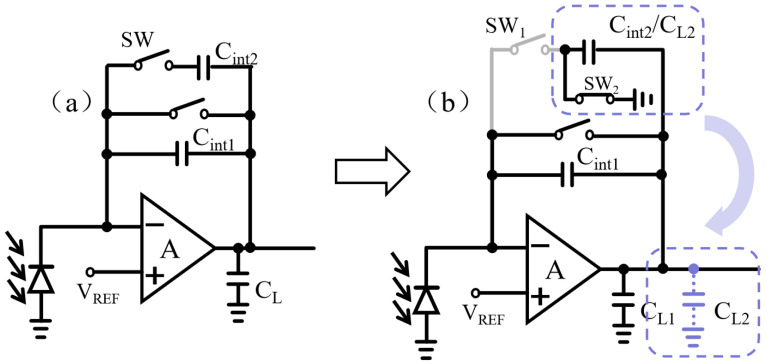
Short-wave infrared ROIC input stage structure: (**a**) traditional CTIA input stage; (**b**) input stage structure based on capacitor reuse.

The prior noise analysis of the readout circuit suggests that augmenting the load capacitance is potentially an effective strategy for noise reduction. In standard pixel readout circuit designs, the input stage generally utilizes a method in which capacitors are individually assigned to functional modules. The integration capacitors (e.g., C_int1_, C_int2_) and the load capacitor (CL) are configured independently, as shown in Figure 9a. The restricted pixel area hinders substantial increases in the capacitance values of either component, thereby limiting further enhancement of noise performance.

To resolve the trade-off between input-stage noise and pixel area, we introduce a co-optimized design featuring capacitor reuse, as shown in Figure 9b. Its key feature is a control switch at the input of the primary integration capacitor, C_int1_. When C_int1_ is not engaged in integration, this switch redirects it to the output node, converting it into an auxiliary load capacitor. The operation of the capacitor multiplexing structure, as illustrated in Figure 9b, is managed collaboratively by the gain selection logic and the timing controller. Its core principle is that within any given frame cycle, capacitor C_int2_ assumes only one role at a time—either as an integrating capacitor or as a load capacitor—with its role being determined and switched during the reset period between frames. When the system configuration selects C_int1_ for integration, switch SW_1_ opens while SW_2_ closes. At this point, C_int2_ is effectively connected to the output terminal, functioning as an additional load capacitance C_L2_. The states of switches SW_1_ and SW_2_ are set only at the commencement of the frame reset phase and remain constant throughout the subsequent integration and readout phases to prevent the introduction of switching transient noise. By employing a transfer gate switch with complementary clock signals and precisely dimensioning the switching transistors, charge injection and clock crosstalk effects introduced by switching are minimized. During the reset phase at the start of each frame, regardless of C_int2_’s subsequent role, its plates are reset to a known potential *V*_REF_, eliminating any residual charge that may remain on the capacitor.

This reuse strategy offers a significant advantage: it substantially increases the effective load capacitance without requiring an additional layout area. Consequently, it lowers the input-referred noise. This approach thus improves both noise performance and area efficiency within the stringent constraints of a small pixel.

## 4. Readout Integrated Circuit for IRFPA

### 4.1. ROIC on the Chip

**Figure 10:**
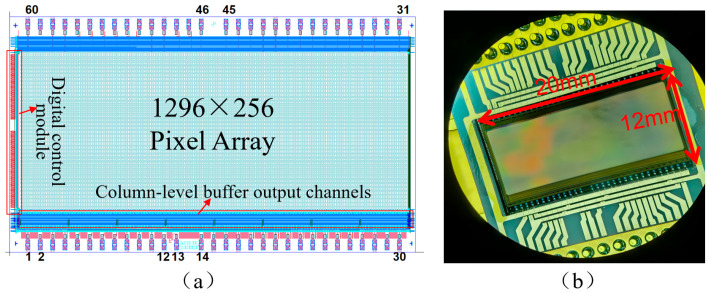
(**a**) Layout of the ROIC. (**b**) A microscope photo of the proposed IRFPA.

A specialized readout integrated circuit (ROIC) for infrared focal plane arrays (FPAs) has been built, utilizing the pixel-level single-ended CDS architecture and capacitor reuse technology suggested in this study. The circuit has a configuration of 1296 columns by 256 rows and a pixel pitch of 15 µm, produced using a conventional CMOS process. The overall layout dimensions of the ROIC chip are 20 mm × 12 mm. Figure 10a illustrates the comprehensive circuit configuration, which primarily comprises a pixel array, a digital control module, column-level buffers, and a multi-channel parallel output interface. The ROIC was subsequently flip-chip connected to an InGaAs short-wave infrared photodetector array to create a complete IRFPA. A microscopic image of the manufactured IRFPA is shown in Figure 10b.

### 4.2. Results and Analysis

**Figure 11:**
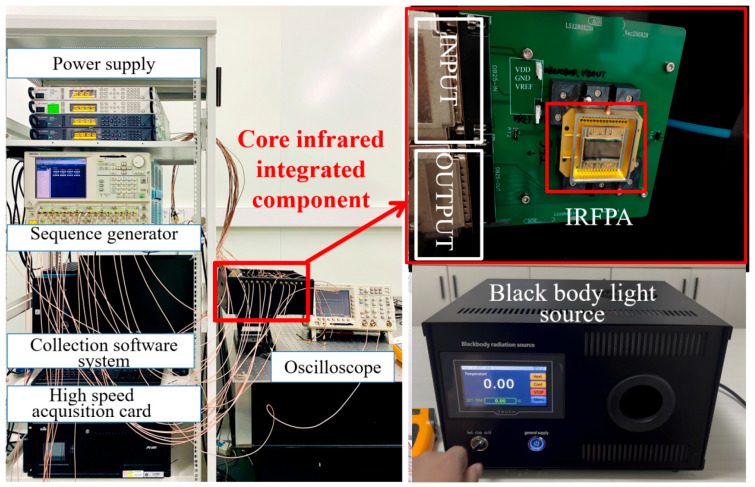
The specialized testing system for IRFPA.

Figure 11 depicts the establishment of a specialized test system to evaluate the performance of the infrared focal plane array (FPA) module. The system comprises a power supply module, a timing generator, a data acquisition system, a high-speed acquisition card, and a blackbody radiation source. All measurements were performed at ambient temperature (25 °C).

**Figure 12:**
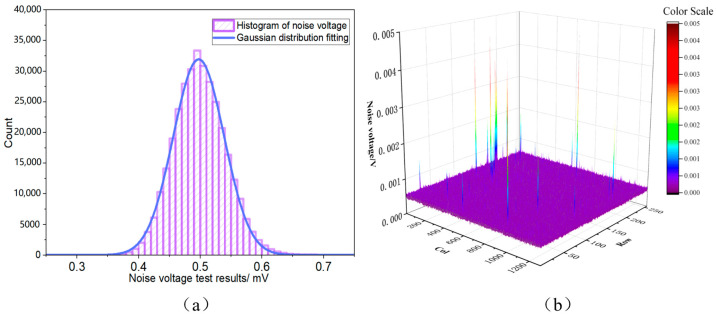
(**a**) Histogram of Noise Voltage Distribution. (**b**) Three-dimensional noise distribution of IRFPA.

To precisely assess the noise performance of the engineered readout circuit, extensive noise testing was performed under high-gain mode circumstances, employing an integration capacitance of 12 fF. In total darkness, 100 sequential frames of dark noise output signals were recorded for statistical analysis. In order to precisely characterize the intrinsic random noise of the readout circuitry, we conducted time-domain noise measurements under full-dark conditions. The tests were conducted at room temperature (25 °C) with the chip powered by AVDD at 3.3 V and DVDD at 1.8 V, operating in IWR mode. Under an integration time of 1 ms and an integration capacitance of 12 fF, 100 consecutive frames of dark-field output voltage data were acquired. The root mean square error was then calculated for each pixel to determine its time-domain noise voltage. The median noise value of 0.5 mV across all active pixels was adopted as the circuit’s noise voltage. At this operating point, with the specified integration capacitance, the conversion gain was 13.3 μV/e^−^, and the equivalent noise charge (ENC) was 37.6 e^−^. Figure 12a illustrates the histogram of the noise voltage distribution. The noise voltage distribution approximates a Gaussian distribution, with the peak voltage corresponding to approximately 0.5 mV aligning closely with the calculated median noise voltage. This indicates a concentrated and symmetrical noise distribution. Figure 12b depicts the three-dimensional noise distribution throughout the whole focal plane array. Aside from a few problematic pixels displaying increased noise levels, the overall pixel noise remains predominantly within the 0.5 mV range, indicating adequate spatial uniformity.

**Figure 13:**
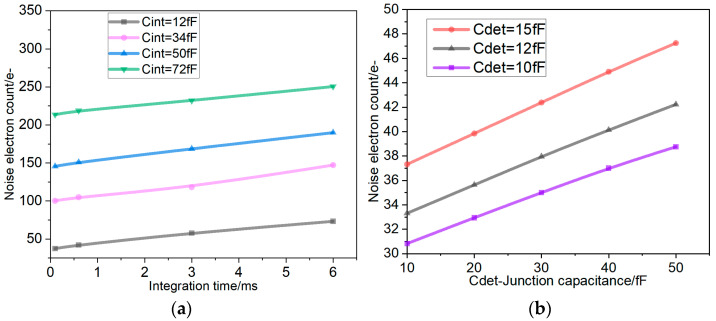
(**a**) Measured circuit noise variation with integration time. (**b**) Simulation effect of detector junction capacitance on noise.

Additionally, this design offers four adjustable gain settings corresponding to different integration capacity values. To systematically evaluate the noise performance of this design, the variation of noise with integration time was tested across each integration capacity setting, as illustrated in Figure 13a. As *C*_int_ increases, the conversion gain decreases, the full-well capacity rises, and the equivalent noise electron count exhibits an upward trend due to thermal noise effects, consistent with noise theory. As indicated by Equations (5)–(8), the circuit’s input reference noise is also influenced by the detector junction capacitance *C*_det_. Since the detector junction capacitance is fixed in this circuit, Figure 13b simulates the trend of equivalent noise electrons with varying junction capacitance *C*_det_ at a fixed gain. It is evident that a smaller detector junction capacitance results in fewer equivalent noise electrons for the circuit.

**Figure 14:**
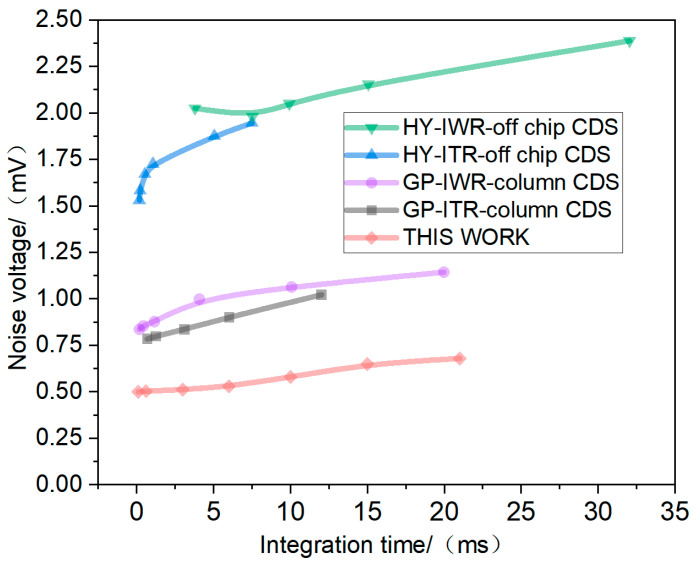
Noise levels for tested conditions under different intergration times.

To evaluate the noise performance of our design, we compared it with several earlier readout circuits from our group. These included conventional off-chip CDS differential architectures and column-level CDS designs, operating in both IWR and ITR modes. All noise measurements were conducted in a completely dark environment, with the detector biased at its typical operating point. The circuits employed for comparison in this work adopted identical or highly similar circuit architectures, including column-level buffers, output multiplexers, and output drivers. The layout of these modules was maintained consistent through collaborative design. Furthermore, all noise data was acquired using the same high-precision test system. The comparison results are shown in Figure 14. All comparative tests were conducted under matched conditions, with corresponding working integration capacitances of 12 fF or equivalent levels. Under identical IWR mode with an integration time of 6 ms, the single-ended output 1296 × 256 circuit proposed herein achieved approximately 70% noise voltage reduction compared to the conventional off-chip CDS architecture.

This reduction directly addresses the high noise typically associated with traditional IWR operation. The result validates a key advantage of our architecture: its effectiveness in suppressing noise within a compact pixel design.

**Figure 15:**
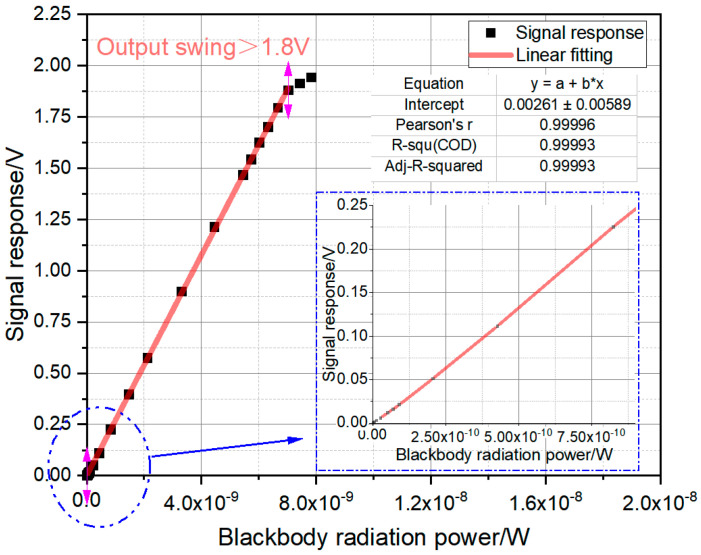
Linearity of incident light intensity response.

We evaluated the circuit’s linearity by measuring its output voltage under different incident light intensities. Figure 15 plots the resulting signal response as a function of input optical power. A linear regression analysis of this curve yields a coefficient of determination (R^2^) greater than 0.9999, confirming an excellent linear fit. Furthermore, the circuit achieves an output swing of over 1.8 V, demonstrating a wide linear dynamic range.

**Figure 16:**
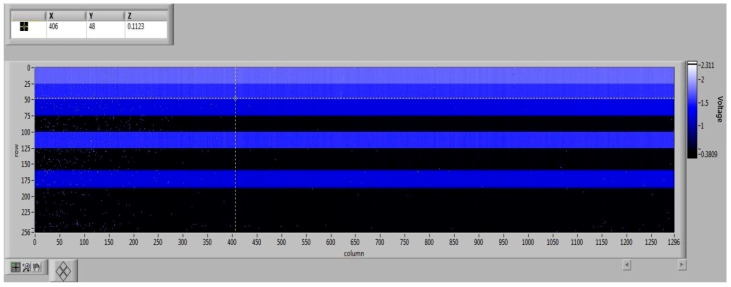
Imaging demonstration of row-level independent adjustment, gating, and gain state selection function.

Furthermore, the circuit supports both row-selection switching and programmable gain for each row. Figure 16 presents the results of an imaging functionality test. By independently adjusting the gain and selection state of different rows, we achieved flexible control over the output image. This programmability is particularly advantageous for hyperspectral imaging applications.

### 4.3. Discussion

A benchmarking analysis comparing our work with analogous ROIC systems, both domestically and globally, is presented in Table 1. The findings demonstrate that the current design attains an advantageous balance between noise performance and integration density, providing a feasible solution for low-noise, high-resolution infrared imaging applications.

The proposed single-ended CDS architecture achieves both compact footprint and low noise, with its robustness being critical for practical applications. This paper primarily focuses on verifying the core functionality and demonstrating typical performance of the new architecture. Comprehensive wide-temperature testing and validation of stability against process variations will be the core of the next phase of research.

## 5. Conclusions

This study proposes a novel ROIC architecture designed to address the trade-off between noise performance and integration density. The work focuses on SWIR focal plane arrays that operate under stringent constraints of small pixel pitch and high-resolution requirements. A high-performance readout solution at 15 µm pixel pitch was achieved by proposing a pixel-level single-ended CDS architecture integrated with capacitor reuse technology. A 1296 × 256 readout circuit was constructed and evaluated. Experimental results indicate that the new readout circuit attains a 70% reduction in noise, achieving a low noise level of 0.50 mV in IWR mode, while maintaining linearity above 0.9999. These performance metrics demonstrate considerable improvement compared to the traditional differential output architecture. The proposed method provides a viable technical solution for balancing the noise–area trade-off in infrared readout circuits, establishing an essential foundation for the development of next-generation high-resolution, compact short-wave infrared imaging systems.

## Figures and Tables

**Table 1:** Performance comparison with previous works.

	Reference [5]	Reference [18]	Reference [19]	This Work
Format	32 × 32	512 × 256	640 × 512	1296 × 256
Process	180 nm	500 nm	500 nm	180 nm
Pixel size	25 µm × 25 µm	30 µm × 30 µm	30 µm × 30 µm	15 µm × 15 µm
Support voltage	3.3 V/1.8 V	3.3 V/1.8 V	5.5 V	3.3 V/1.8 V
Read architecture	Pixel-level CDS	Pixel-level CDS	Pixel-level CDS	Pixel-level CDS
Read mode	IWR, ITR	IWR, ITR	IWR, ITR	IWR, ITR
Linearity	/	/	>99.9%	>99.9%
Noise electron */(IWR)	143e-@30 fF	160e-@70 fF	129e-@30 fF	37.6e-@12 fF
Power consumption	200 mW	150 mW	150 mW	200 mW
Frame rate	/	/	350 Hz	>200 Hz

* Mainly focusing on noise voltage in IWR mode.

## Data Availability

Data are contained within the article.
